# Gut Microbiome Characteristics in Mothers and Infants According to the Presence of Atopic Dermatitis

**DOI:** 10.1155/2022/8145462

**Published:** 2022-04-23

**Authors:** Myongsoon Sung, Yujin Choi, Hyunjoon Park, Chul Sung Huh

**Affiliations:** ^1^Department of Pediatrics, Soonchunhyang University Gumi Hospital, Gumi, Republic of Korea; ^2^Department of Pediatrics, CHA Gumi Medical Center, CHA University School of Medicine, Gumi, Gumi, Gyeongsangbuk-do, Republic of Korea; ^3^Research Institute of eco-Friendly Livestock Science, Institute of Green-bio Science and Technology, Seoul National University, Pyeongchang, Republic of Korea; ^4^Graduate School of International Agricultural Technology, Seoul National University, Pyeongchang, Republic of Korea

## Abstract

**Objective:**

The role of the gut microbiome in the onset and development of atopic dermatitis (AD) has been postulated. Thus, we investigated the gut microbial compositions in infants with and without AD and compared the gut bacterial flora of their mothers.

**Methods:**

The prospective and cross-sectional study participated in 44 pairs of mothers and children. We selected infants born via full-term normal vaginal delivery and no history of antibiotic or probiotic use and infection during the first three months of life. The 15 pairs, consisting of nine healthy infants and six AD infants, were included in this study. Fecal samples of mothers and infants were analyzed within 30 days of delivery and at 12 months, respectively. Microbes in the fecal samples of mothers and infants were subjected to analysis of 16S rRNA amplicon sequencing.

**Results:**

The abundance of specific taxonomic groups was notably different, but microbial diversity and phylogenetic distances were not significantly different in either maternal or infant groups according to the presence of infant AD. A total of 12 species were selected as differential species in infants with AD compared to healthy infants. Six species were significantly different in the mothers of infants with AD compared to the mothers of healthy infants. *Akkermansia muciniphila* was only detected in healthy infants and their mothers.

**Conclusion:**

The presence of *Akkermansia muciniphila* in mothers and children after vaginal delivery is associated with the onset and development of AD.

## 1. Introduction

Atopic dermatitis (AD) is one of the most common types of dermatosis in infants [1]. AD in children affects 17-24% of the total pediatric population and develops during the first six months of life in 45% of the affected children and by five years of age in 85% of those affected [1]. However, the underlying pathology of AD is heterogeneous, and causes are known to be a poorly defined mix of innate and adaptive immune responses [2].

Meanwhile, previous studies have shown that the gut encompasses diverse and dynamic microbial ecology linked to human health and various diseases [3]. Since the role of the gut microbiome in the early development of the immune system has been reported, its impact on allergic diseases, including AD, has been explored [4]. Additionally, the first 12 months of life is a critical period of microbial colonization in the gastrointestinal tract [5]. So, recent studies have shown the differences in the gut microbiome of infants with AD and those without AD [5–7]. Moreover, temporal relationships of the gut microbiota with multisensitized atopy and IgE-associated eczema have been reported [7]. Various factors affect the microbial colonization of the infants' gut, such as the delivery mode, feeding method, weaning period, regular diet, probiotics consumption, and antibiotic treatments [8, 9]. Furthermore, these factors may influence the development of AD in infants [10–12]. The perturbations of the mother's gut microbiome may be associated with the poor development of the infant's immune system and a higher risk of AD [11, 12].

In the present study, we hypothesized that the specific status of the mother's gut microbiome might be associated with the development of the immune system and the risk of AD in infants. Additionally, AD's natural course in infants is characterized by development during an early stage, from the first six months to 12 months. Thus, we aimed to investigate the mother and infant-associated early microbiome markers to predict AD in infants.

## 2. Materials and Methods

### 2.1. Subjects

This prospective cross-sectional study enrolled maternal and child pairs between December 2016 and December 2018. The study involved the SCORing Atopic Dermatitis (SCORAD) score assessment at six and 12 months of age and skin prick testing (SPT) and fecal sampling at 12 months of age for the infant. Also, fecal samples from the mother were collected within 30 days of delivery. A total of 44 pairs of mothers and children provided their consent to participate in the study. We selected infants born via full-term normal vaginal delivery and who had no history of antibiotic or probiotic use and infection during the first three months of life. They were fed with a combination of breast milk and formula or breast milk for the first six months of life and started solid food intake within six months of age. Additionally, the mothers did not consume any probiotics during pregnancy, and all families lived with no pets. Infants and mothers had no history of antibiotic use before fecal sampling for seven days. Eventually, this study population consisted of 15 maternal and child pairs from nine healthy infants and six AD infants.

The diagnosis of allergic disease (AD, allergic rhinitis, and asthma) was made based on the Korean ISAAC questionnaire, a standardized method of evaluating allergic diseases in epidemiologic studies in Korea [13]. Following the criteria of Hanifin and Rajka [14], a pediatrician made the diagnosis of AD after a physical examination of each child and calculated the SCORAD scores at six and 12 months of age. The AD group was divided into three classes based on the severity of AD: mild (<25), moderate (25-50), and severe (>50) [15].

In the present study, the infant AD group was identified through a comprehensive evaluation of clinical history, including lifetime AD symptoms via questionnaire and physical examination by a pedestrian specializing in allergies at six and 12 months of age. The healthy infant control group consisted of children who had a SCORAD score of 0 on physical examination by the same pediatrician and no history of allergic diseases (AD, allergic rhinitis, or asthma) as assessed in the questionnaire during the same period.

### 2.2. Measurements

SPT was performed with standardized allergen extracts and control solutions from LaForma (Milan, Italy) on the volar surface of both arms. Subjects were tested for sensitivity to the common aeroallergens: house dust mites (HDM; *Dermatophagoides pteronyssinus (D.p)* and *Dermatophagoides farina (D.f)*) and four common food allergens (eggs, milk, peanut, and soy). SPT was performed on infants with AD between12 and 13 months of age, and a positive SPT was defined as one with a mean wheel diameter that was 3 mm or more significant than the positive control. Atopy was defined as the presence of positive SPT. Fecal samples were collected from mothers within 30 days of childbirth and their infants at 12 months. Thirty fecal samples were collected from nine pairs of non-AD healthy infants (IHC group) and their mothers (MHC group) and six pairs of AD infants (IAD group) and their mothers (MAD group). The samples were obtained in bottles containing DNA/RNA Shield (Zymo Research, Irvine, CA, USA) at an equal volume (w/v) and stored immediately at –80 °C. According to the manufacturer's instructions, cellular DNA extraction was conducted using the ZymoBIOMICS DNA Miniprep Kit (Zymo Research).

### 2.3. Miseq 16S rRNA Amplicon Sequencing and Microbiome Analysis

Sequencing libraries of the V3–V4 regions of the 16S bacterial rRNA gene were constructed following Illumina's protocol [16]. Two-step PCR with two clean-up procedures was conducted, and the obtained 16S rRNA amplicons were sequenced on the Illumina MiSeq platform (Illumina Inc., San Diego, CA, USA) by ChunLab Inc. (Seoul, South Korea). Detailed amplicon and index PCR conditions were as follows: amplicon PCR (95 °C for 3 min; 25 cycles of 95 °C for 30 s, 55 °C for 30 s, and 72 °C for 30 s; and 72 °C for 5 min) and index PCR (95 °C 3 min; 8 cycles of 95 °C 30 s, 55 °C 30 s, and 72 °C 30 s; and 72 °C for 5 min).

In the Quantitative Insight into Microbial Ecology 2 (QIIME 2) pipeline [17], the paired-end sequenced reads were demultiplexed and quality controlled using the DADA2 algorithm to obtain an amplicon sequence variant (ASV) table. Phylogenetic trees were created, and taxonomy was annotated with the naive Bayes classifier against the Greengenes database. The obtained files were imported into R software (http://www.r-project.org) and transformed to phyloseq objects for the subsequent analysis [18]. Chao1 and Shannon indices were applied to measure microbial richness and diversity. Weighted-UniFrac and generalized UniFrac methods were used, and the sample distribution was determined using the MDS/PCoA and NMDS methods [19]. To determine whether group separations were significant, ADONIS and permutational MANOVA (PERMANOVA) tests were conducted. The differential abundances of specific microbial taxa were analyzed using the DESeq2 package [20].

### 2.4. Ethics

This study protocol was approved by the Institutional Review Boards (IRBs) of the CHA Gumi Medical Center (IRB No. 2016-1137). Written informed consent was obtained from the parents or guardians of all participants following a detailed explanation of the study.

### 2.5. Statistical Analysis

SPSS Statistics ver. 19.0 (IBM Co., Armonk, NY, USA) was used for all statistical analyses. Values are reported as the mean ± standard deviation. Scale variables were analyzed using Fisher's exact test, and continuous variables were analyzed using the Mann–Whitney *U*-test. A correlation map was constructed using the MeV software package (http://www.tm4.org/) based on correlation coefficient values calculated using PASW Statistics 18 software. Statistical significance was defined as *P* < 0.05. Relative risk (RR) ratios and corresponding 95% CIs were calculated using log-binominal regression with the maximum likelihood estimation in R software.

The significance of the relative abundances was analyzed with a two-tailed *t*-test to evaluate differences in samples of discrete variables in GraphPad Prism (version 8.3.0; GraphPad software Inc., San Diego, CA, USA).

## 3. Results

### 3.1. General Characteristics

Fifteen pairs of infants and mothers participated in the study: nine in the HC group and six in the AD group (Table 1). All infants had a full-term normal vaginal delivery and no history of antibiotic or probiotic use and infection during the first three months of life. They started solid-food intake within six months old. They were fed with breast milk or a combination of breast milk and formula for the first six months, but the ratio of breast milk and breast milk and formula were different (5 : 4 in HC group/5 : 1 in AD group). There was no significant difference between the HC and AD groups concerning breastfeeding duration or mixed feeding. The overall severity of AD was mild and moderate-severe in five infants (83.3%) and one infant (16.7%) in the AD group, respectively. Among the six infants with AD, three infants had sensitization to allergens, and the most common allergen was eggs.

The mother subjects had no probiotic consumption during pregnancy, and all families lived without pets (Table 1). There was no statistically significant difference between the HC and AD groups concerning allergic diseases and diet in pregnancy. Two mothers had a history of antibiotic use during pregnancy in the AD group, but there was no statistically significant difference between the two groups (*P* = 0.143).

### 3.2. Maternal Gut Bacterial Differences Based on Child AD Diagnosis

**Figure 1:**
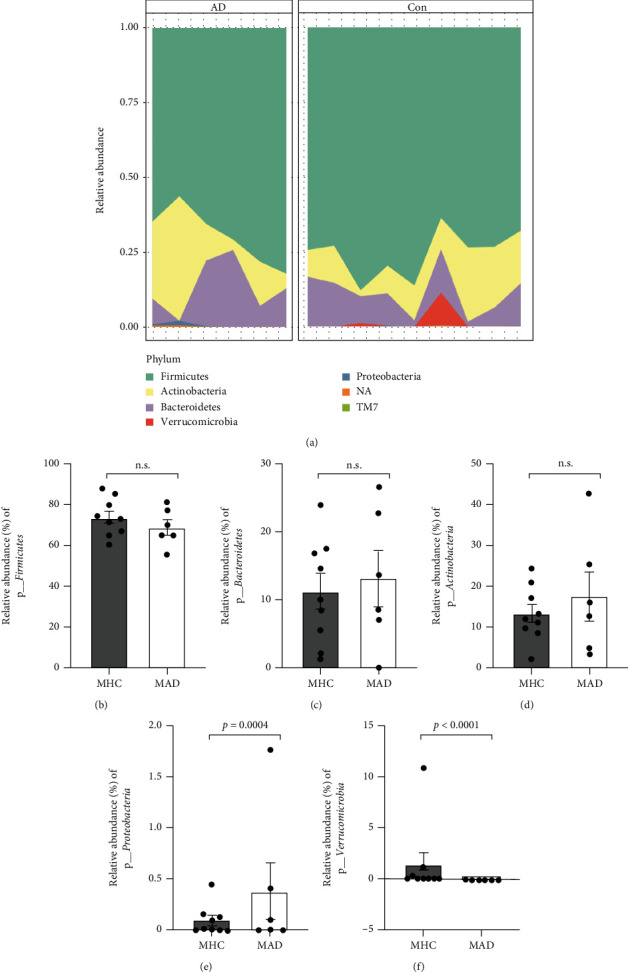
Phylum relative abundances comparison of maternal gut microbiome. (a) Phylum level. (b) *Firmicutes*. (c) *Bacteroidetes.* (d) *Actinobacteria*. (e) *Proteobacteria*. (f) *Verrucomicrobia.*

**Figure 2:**
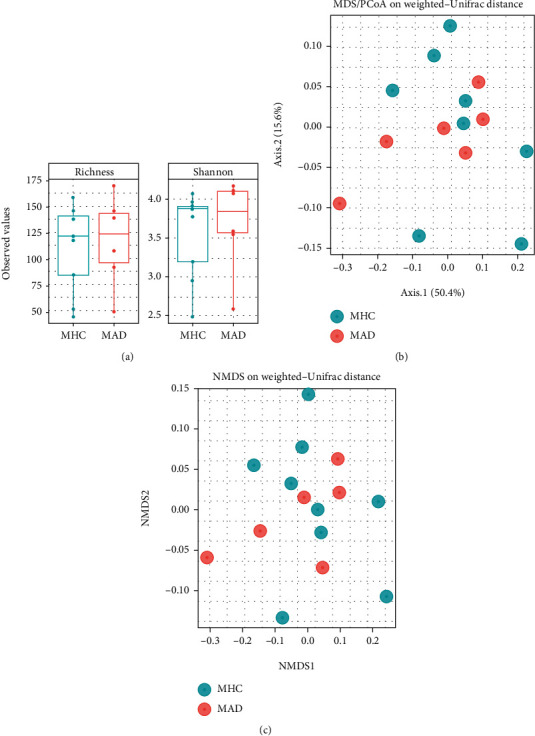
Analysis of maternal groups. (a) Bacterial composition with regard to Chao1 and Shannon diversity between maternal groups. (b, c) In beta diversity analysis, sample distributions in the maternal groups showed partial differences without significance (*F* value = 0.852, R squared = 0.051) .

At the phylum level, there were changes in gut microbial composition of maternal individuals (Figure 1(a)). Both mother groups showed high populations of *Firmicutes* in the gut (Figure 1(b)). The relative abundances (RAs) of *Bacteroidetes* and *Actinobacteria* were similar between the maternal groups (Figures 1(c) and 1(d)). The population of *Proteobacteria* was significantly higher in the MAD group (0.37%) than in the MHC group (0.09%) (*P* = 0.0004) (Figure 1(e)). Of note, the *Verrucomicrobia* phylum was detected only in the MHC group (RAs = 1.38% and *P* < 0.0001) (Figure 1(f)). However, there were no significant differences in bacterial composition with regard to Chao1 and Shannon diversity between maternal groups (Figure 2(a)). In beta diversity analysis, sample distributions in the maternal groups showed partial differences without significance (*F* value = 0.852, *R* squared = 0.051) (Figures 2(b) and 2(c)).

### 3.3. Gut Bacterial Differences Based on AD Diagnosis

**Figure 3:**
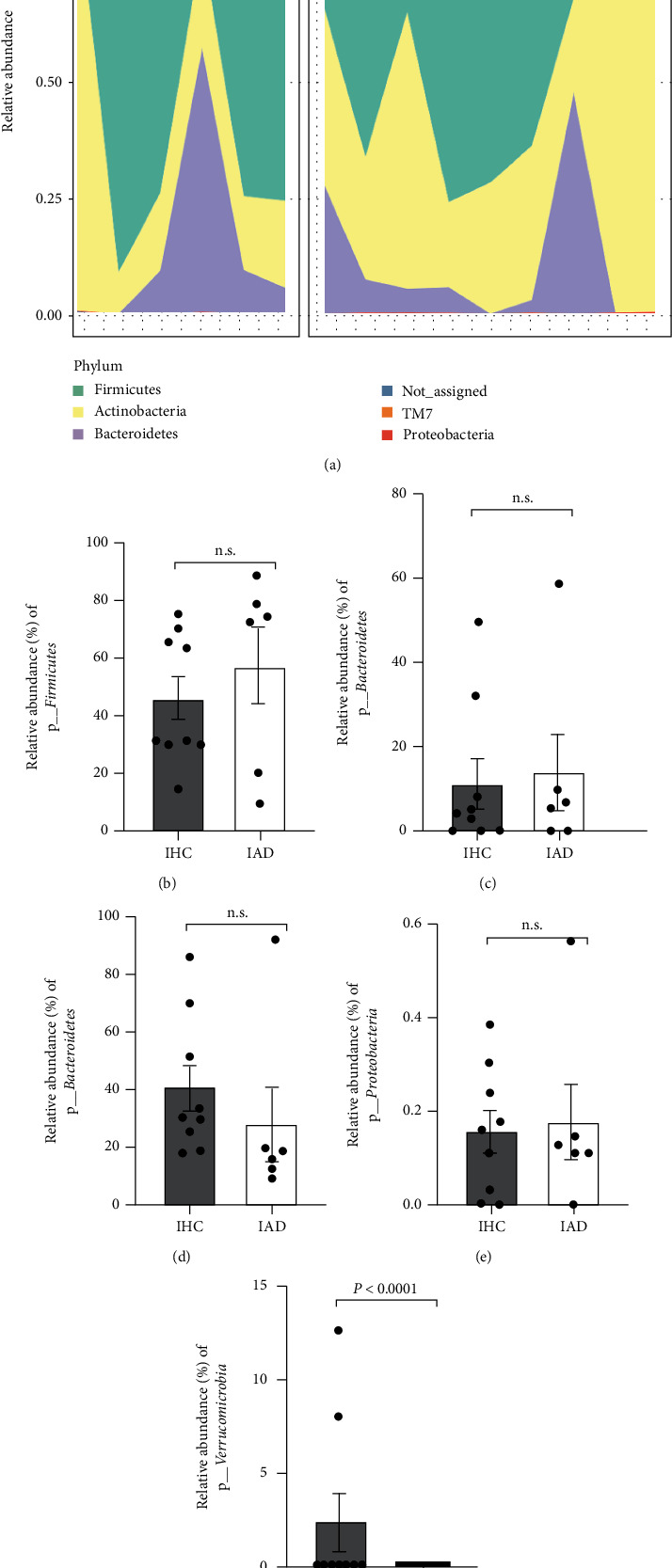
Phylum relative abundances comparison of infant gut microbiome. (a) Phylum level. (b) *Firmicutes*. (c) *Bacteroidetes.* (d) *Actinobacteria*. (e) *Proteobacteria.* (f) *Verrucomicrobia.*

**Figure 4:**
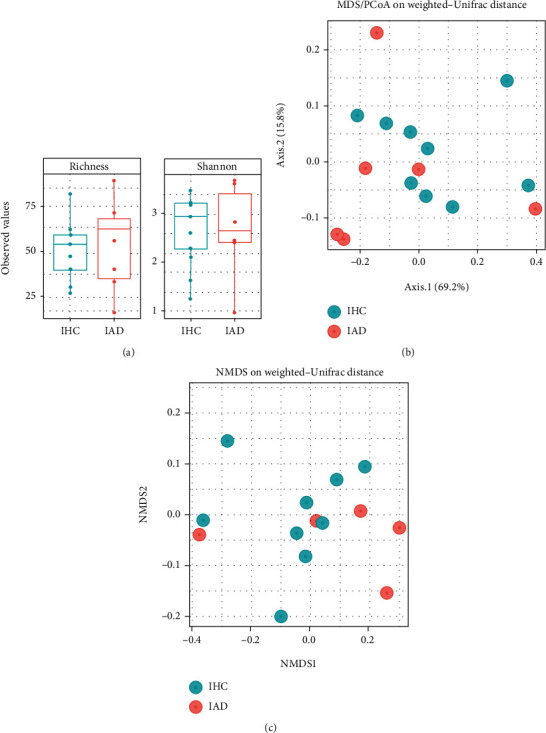
Analysis of infant groups. (a) Bacterial composition with regard to Chao1 and Shannon diversity between infant groups. (b, c) Considerable phylogenetic separation was not observed in beta diversity analysis (*F* = 0.592, R squared = 0.057).

The characteristics of the infant gut microbial composition are shown in Figures 3 and 4. A total of 15 infants were included, comprising nine healthy controls and six AD cases, and they showed individual patterns of the gut microbiome (Figure 3(a)). Among the assigned phyla, *Firmicutes*, *Bacteroidetes*, and *Actinobacteria* were predominant in all infant (Figures 3(b)–3(d)). The IHC group showed relatively higher abundances of *Actinobacteria*, but the difference was not statistically significant (*P* = 0.4614) (Figure 3(d)). There was no considerable difference in the RAs of *Proteobacteria* between the infant groups (Figure 3(e)). In particular, the population of *Verrucomicrobia* was detected only in the IHC group (2.312%) (*P* < 0.0001) (Figure 3(f)). The infant groups showed no significant differences in the alpha (Figure 4(a)) and beta diversity (Figures 4(b) and 4(c)), and considerable phylogenetic separation was not observed (*F* = 0.592, *R* squared = 0.057).

### 3.4. Differential Abundance in Specific Microbial Taxonomic Group

**Figure 5:**
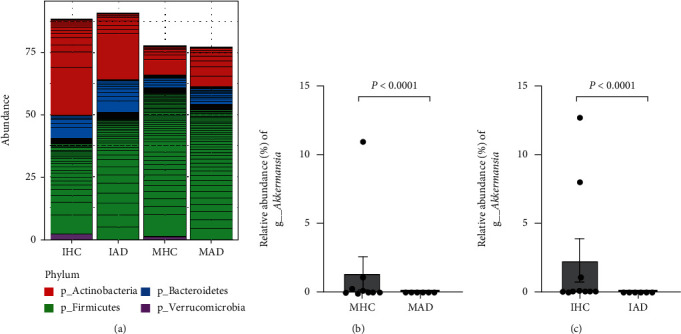
The most abundant bacterial taxa applied by the DESeq2 in each group. (a) Phylum level. (b) Relative abundance of *Akkermansia* in mother groups. (c) Relative abundance of *Akkermansia* infant groups.

In our results, there were no notable differences between groups in microbial richness/diversity and the RAs at the phylum level (Figures 1 and 3), except for *Verrucomicrobia* (Figures 1(f) and 3(f)) and *Proteobacteria* (Figure 1(e)). Thus, we sorted the top 100 most abundant bacterial taxa (Figure 5(a)) and applied the DESeq2 method to find differential microbial taxa between groups using statistical criteria (BaseMean > 1 and *P* < 0.05) (Table 2). In the infant group comparison, seven operational taxonomic units (OTUs) (classified as *Bifidobacterium*, *B. breve*, *Clostridium paraputrificum*, uncultured *Clostridiales*, uncultured *Lachnospiraceae*, and *Akkermansia muciniphila*) were significantly lower in the IAD group than in the IHC group, while five OTUs (*Bacteroides*, *Dorea longicatena*, *Faecalibacterium*, and *Ruminococcus lactaris*) were significantly higher in the IAD group. In the case of the maternal groups, *Coprococcus eutactus*, *Ruminococcus lactaris*, uncultured *Clostridiales*, and *Akkermansia muciniphila* were significantly lower in the MAD group than in the MHC group, but only *Prevotella* was a differentially higher taxon in the MAD group. Among the selected taxa, *Akkermansia*, which belongs to the phylum *Verrucomicrobia*, showed similar patterns in the maternal and infant groups based on the presence of AD in children (Figures 5(b) and 5(c)).

## 4. Discussion

In the present study, we compared the composition and genes of the gut microbiome in healthy infants and those with AD at 12 months of age and their mothers within 30 days of birth. Interestingly, we demonstrated that *Akkermansia muciniphila* (*A. muciniphila*) was present in the gut microbiome of healthy children in early life and their mothers, but not in children with AD and their mothers which supports the previous findings of the gut microbiome's role in the onset of AD. It has been established that the maturation of the gut microbiome from prepartum to three years of age is influenced by various maternal determinants, such as host genetics, feeding method, maternal diet, maternal infections, and delivery mode [21, 22]. These results postulated that the difference in the gut microbiome according to the infant's AD might be derived from the mother's gut flora. Thus, we analyzed whether the pattern of gut microbial composition in the mother groups was similar to the composition detected in the infant groups.

*A. muciniphila*, which was first isolated in 2004 [23], is a probiotic species associated with human health and diseases, such as obesity, type 2 diabetes, and colorectal cancer [24]. Some recent studies demonstrated the absence of *A. muciniphila* in the gut microbiome of children with AD [25], which might play a role in IgE-mediated atopic disease. Moreover, *A. muciniphila* contributes to mucosal innate immune response regulation due to its mucus-degrading characteristics [25]. According to a recent study, the proportion of *A. muciniphila* was higher in children with transient AD than in children with non-AD or persistent AD [26]. However, in our study, the proportion of *Akkermansia* was higher in children with AD than in children without AD because we included only the persistent AD subtype. Thus, more researches are needed to clarify these relationships [26].

Meanwhile, the reduced abundance of some taxa, including *Bifidobacterium*, *Clostridium*, *Lachnospiraceae*, and *Faecalibacterium*, has been commonly reported in children with AD [21, 27], which is in agreement with the present study, even though there has been an ongoing debate about gut microbiomes associated with allergic disease. It is well known that *Bifidobacterium*, one of the major genera in an infant's gut, is less abundant in infants with AD [21, 27], following the present findings. However, some researchers suggest that oligosaccharide type and composition affect the composition of *Bifidobacterium* spp. in the gut according to the feeding method and thus lead to the development of atopic disorders [21, 27, 28]. However, this data is different from our data, in which all infants had breast milk feeding or mixed feeding, but *Bifidobacterium* was significantly lower in the children with AD. The populations of *Faecalibacterium* and *Lachnospiraceae* in infants with AD changed inversely in a longitudinal study of the microbiome of infants with up to 200 days of age [29].

Some studies suggested that maternal diet and infection during pregnancy influence the infant's gut microbiome [21, 22, 29]. The perturbation process results in changes in the host-microbiome biodiversity and metabolic activities. It has been associated with greater susceptibility to immune-mediated disorders, such as AD, later in life [22, 29]. In the present study, in the MAD group, two mothers had antibiotic medication during pregnancy, but there was no significant difference between the MAD and MHC groups, probably due to the small sample size. The sample size used in this analysis was relatively small because of the exclusion criteria applied (normal delivery with full term, breast milk feeding, and no antibiotic medication for the first three months). Therefore, we need to be careful with the generalization of these results and more mother and infant combination data.

The AD severity was mostly mild, as our subjects were sourced from a general population-based prospective cohort study not patient with AD. Additionally, the human intestinal microbiota comprised of bacteria and fungi, but we did not consider the roles of fungi. Further studies are necessary to resolve these limitations, including a replication study using most of our current subjects and functional studies to assess these phenomena mechanistically.

However, this study has several strengths. First, our study subjects were recruited from a prospective cohort study. Second, we analyzed stool samples from children who had not received antibiotics for the first three months and mothers and infants with no history of antibiotic usage before seven days of fecal sampling. The history of antibiotic usage could have affected the composition of the gut microbiome and the relationship between mother-child gut microbiomes. Third, the AD phenotype, in terms of the natural course of this disorder, was assessed by the same pediatric allergist twice. We analyzed the follow-up data regarding the progression of AD, which was recorded by a pediatric allergist from birth to 12 months of age, and performed 16s rRNA sequencing.

## 5. Conclusions

We found an abundance of *Akkermansia* only in healthy maternal-child pairs among the taxa analyzed, but not in infants with AD and their mothers. Based on previous studies, we assume that the less abundant *Akkermansia* in infants with AD may be derived from their mother's gut flora, which may have affected the onset or development of AD in infants. In conclusion, the gut microbiome and its influence on innate immune development in infants and mothers play a crucial role in infants with AD. Further studies are needed to identify the association and roles of *Akkermansia* in the infant gut and development of atopic disorders.

## Figures and Tables

**Table 1:** Demographic and clinical characteristics of the total study population.

Variable	Healthy controls *n* = 9 (100)	AD *n* = 6 (100)	*P* value
*Infant*			
Male gender, *n(%)*	7 (77.8)	2 (33.3)	0.136
Parental allergic diseases, *n(%)*	6 (66.6)	6 (100)	0.229
Administration of antibiotics, *n(%)*	5 (55.5)	5 (83.3)	0.580
<3 months	0	0	
3–5 months	2	2	
≥6 months	3	3	
Breast-feeding or mixed feeding, *n(%)*			1.000
≤6 months	7 (77.7)	4 (66.6)	
>6 months	2 (22.3)	2 (33.4)	
Presence of sibling (yes), *n(%)*	5 (55.5)	5 (83.3)	0.580
Onset time of AD, *n(%)*			
≤6 months	NA	3 (50.0)	
>6 months	NA	3 (50.0)	
SCORAD of 12 months	NA	13.5 ± 7.17	
Severity of AD at 12 months, *n(%)*			
Mild (<25)	NA	5 (83.3)	
Moderate (25 ≤ and ≤50) and severe (>50)	NA	1 (16.7)	
Skin prick test result, *n(%)*	NA	3 (50.0)	
Egg, *n* (%)	NA	2 (33.3)	
HDM, *n* (%)	NA	1 (16.7)	
*Mother*			
Mean age	33.33 ± 3.39	30.66 ± 4.58	0.236^∗^
Allergic diseases, *n(%)*	2 (22.2)	4 (66.6)	0.136
Antibiotics treatment in pregnancy, *n(%)*	0	2 (33.3)	0.143
Diet in pregnancy, *n (%)*			0.608
Vegetarian or Korea traditional diet	5 (55.5)	2 (33.3)	
Meat or instant diet	4 (44.5)	4 (66.7)	

Values are presented as number (%) and mean ± standard error. AD, allergic dermatitis; NA, not applicable; SCORAD, scoring of atopic dermatitis. HDM, house dust mite. ∗Mann–Whitney test.

**Table 2:** Differential microbial taxa in group comparisons determined using DESeq2.

Compared groups	BaseMean	log2FoldChange	*P* value	Phylum	Species
IAD over IHC	29.46	-8.33	0.0024	*Actinobacteria*	*Bifidobacterium*
	34.72	-22.91	<0.001	*Actinobacteria*	*Bifidobacterium*
	861.94	-4.03	0.0021	*Actinobacteria*	*Bifidobacterium breve*
	14.16	23.05	<0.001	*Bacteroidetes*	*Bacteroides*
	5.79	6.12	0.0432	*Bacteroidetes*	*Bacteroides*
	5.98	-6.03	0.0430	*Firmicutes*	*Clostridium paraputrificum*
	11.37	22.76	<0.001	*Firmicutes*	*Dorea longicatena*
	6.20	6.22	0.0399	*Firmicutes*	*Faecalibacterium*
	7.95	6.58	0.0297	*Firmicutes*	*Ruminococcus lactaris*
	21.00	-22.21	<0.001	*Firmicutes*	unknown_*Clostridiales*
	8.72	-6.57	0.0319	*Firmicutes*	unknown_*Lachnospiraceae*
	29.54	-22.68	<0.001	*Verrucomicrobia*	*Akkermansia muciniphila*

MAD over MHC	19.65	5.38	0.0417	*Bacteroidetes*	*Prevotella*
	10.11	-6.57	0.0320	*Firmicutes*	*Coprococcus eutactus*
	22.50	-22.00	<0.001	*Firmicutes*	*Ruminococcus lactaris*
	21.23	-7.64	0.0032	*Firmicutes*	unknown_*Clostridiales*
	24.46	-7.84	0.0007	*Firmicutes*	unknown_*Clostridiales*
	7.01	-6.04	0.0488	*Verrucomicrobia*	*Akkermansia muciniphila*

AD, atopic dermatitis, IAD, infant AD; IHC, infant healthy controls; MAD, mother AD; MHC, mother healthy controls. Criteria for inclusion: mean of normalized counts for all samples (BaseMean) >1 and *P* value <0.05.

## Data Availability

The datasets used and/or analyzed during the current study are available from the corresponding author on reasonable request for clinical research purposes.

## References

[1] Laughter D., Istvan J. A., Tofte S. J., Hanifin J. M. (2000). The prevalence of atopic dermatitis in Oregon schoolchildren. *Journal of the American Academy of Dermatology*.

[2] Shreiner A. B., Kao J. Y., Young V. B. (2015). The gut microbiome in health and in disease. *Current Opinion in Gastroenterology*.

[3] Bäckhed F., Roswall J., Peng Y. (2015). Dynamics and stabilization of the human gut microbiome during the first year of life. *Cell Host & Microbe*.

[4] Wopereis H., Oozeer R., Knipping K., Belzer C., Knol J. (2014). The first thousand days- intestinal microbiology of early life: establishing a symbiosis. *Pediatric Allergy and Immunology*.

[5] Fujimura K. E., Sitarik A. R., Havstad S. (2016). Neonatal gut microbiota associates with childhood multisensitized atopy and T cell differentiation. *Nature Medicine*.

[6] Zheng H., Liang H., Wang Y. (2016). Altered gut microbiota composition associated with eczema in infants. *PLoS One*.

[7] Arrieta M.-C., Stiemsma L. T., Dimitriu P. A. (2015). Early infancy microbial and metabolic alterations affect risk of childhood asthma. *Science Translational Medicine*.

[8] Gupta V. K., Paul S., Dutta C. (2017). Geography, ethnicity or subsistence-specific variations in human microbiome composition and diversity. *Frontiers in Microbiology*.

[9] Zhernakova A., Kurilshikov A., Bonder M. J. (2016). Population-based metagenomics analysis reveals markers for gut microbiome composition and diversity. *Science*.

[10] Milani C., Duranti S., Bottacini F. (2017). The first microbial colonizers of the human gut: composition, activities, and health implications of the infant gut microbiota. *Microbiology and Molecular Biology Reviews*.

[11] Lee E., Kim B.-J., Kang M.-J. (2016). dynamics of gut microbiota according to the delivery mode in healthy Korean infants. *Allergy, Asthma & Immunology Research*.

[12] Rutayisire E., Huang K., Liu Y., Tao F. (2016). The mode of delivery affects the diversity and colonization pattern of the gut microbiota during the first year of infants' life: a systematic review. *BMC Gastroenterology*.

[13] Lee S. I., Shin M. H., Lee H. B. (2001). Prevalences of symptoms of asthma and other allergic diseases in Korean children: a nationwide questionnaire survey. *Journal of Korean Medical Science*.

[14] Hanifin J. M., Rajka G. (1980). Diagnostic features of atopic dermatitis. *Acta Dermato-Venereologica*.

[15] Oranje A. P., Glazenburg E. J., Wolkerstorfer A., de Waard-van F. B. (2007). Practical issues on interpretation of scoring atopic dermatitis: the SCORAD index, objective SCORAD and the three-item severity score. *The British Journal of Dermatology*.

[16] Amplicon P. C. R., Clean-Up P. C. R., Index P. C. R. 16S metagenomic sequencing library preparation. https://www.illumina.com/content/dam/illumina-support/documents/documentation/chemistrydocumentation/16s/16s.

[17] Bolyen E., Rideout J. R., Dillon M. R. (2019). Reproducible, interactive, scalable and extensible microbiome data science using QIIME 2. *Nature Biotechnology*.

[18] McMurdie P. J., Holmes S. (2013). phyloseq: an R package for reproducible interactive analysis and graphics of microbiome census data. *PLoS One*.

[19] Chen J., Bittinger K., Charlson E. S. (2012). Associating microbiome composition with environmental covariates using generalized UniFrac distances. *Bioinformatics*.

[20] Love M. I., Huber W., Anders S. (2014). Moderated estimation of fold change and dispersion for RNA-seq data with DESeq2. *Genome Biology*.

[21] Love M. I., Huber W., Anders S. (2018). Intestinal microbiota in infants at high risk for allergy: effects of prebiotics and role in eczema development. *The Journal of Allergy and Clinical Immunology*.

[22] Peroni D. G., Nuzzi G., Trambusti I., Di Cicco M. E., Comberiati P. (2020). Microbiome composition and its impact on the development of allergic diseases. *Frontiers in Immunology*.

[23] Dubourg G., Cornu F., Edouard S., Battaini A., Tsimaratos M., Raoult D. (2017). First isolation of Akkermansia muciniphila in a blood-culture sample. *Clinical Microbiology and Infection*.

[24] Zhang T., Li Q., Cheng L., Buch H., Zhang F. (2019). Akkermansia muciniphila is a promising probiotic. *Microbial Biotechnology*.

[25] Derrien M., Van Baarlen P., Hooiveld G., Norin E., Müller M., de Vos W. M. (2011). Modulation of mucosal immune response, tolerance, and proliferation in mice colonized by the Mucin-Degrader Akkermansia muciniphila. *Frontiers in Microbiology*.

[26] Park Y. M., Lee S.-Y., Kang M.-J. (2020). Imbalance of GutStreptococcus,Clostridium, andAkkermansiaDetermines the natural course of atopic dermatitis in infant. *Allergy, Asthma & Immunology Research*.

[27] Lee M.-J., Kang M.-J., Lee S.-Y. (2018). Perturbations of gut microbiome genes in infants with atopic dermatitis according to feeding type. *The Journal of Allergy and Clinical Immunology*.

[28] Matsuki T., Yahagi K., Mori H. (2016). A key genetic factor for fucosyllactose utilization affects infant gut microbiota development. *Nature Communications*.

[29] Galazzo G., van Best N., Bervoets L. (2020). Development of the microbiota and associations with birth mode, diet, and atopic disorders in a longitudinal analysis of stool samples, collected from infancy through early childhood. *Gastroenterology*.

